# Tolerance of an Expanding Subarctic Shrub, *Betula glandulosa*, to Simulated Caribou Browsing

**DOI:** 10.1371/journal.pone.0051940

**Published:** 2012-12-13

**Authors:** Emilie Champagne, Jean-Pierre Tremblay, Steeve D. Côté

**Affiliations:** 1 Département de biologie, Université Laval, Québec, Qc, Canada; 2 Centre d'Études Nordiques, Université Laval, Québec, Qc, Canada; University of Helsinki, Finland

## Abstract

Densification of the shrub layer has been reported in many subarctic regions, raising questions about the implication for large herbivores and their resources. Shrubs can tolerate browsing and their level of tolerance could be affected by browsing and soils productivity, eventually modifying resource availability for the caribou. Our objective was to assess the compensatory growth potential of a subarctic shrub, *Betula glandulosa* Michx., in relation with caribou browsing and nutriment availability for the plants. We used a simulated browsing (0, 25 and 75% of available shoots) and nitrogen-fertilisation (0 and 10 g m^−2^) experiment to test two main hypotheses linking tolerance to resource availability, the Compensatory Continuum Hypothesis and the Growth Rate Hypothesis as well as the predictions from the Limiting Resource Model. We seek to explicitly integrate the relative browsing pressure in our predictions since the amount of tissues removed could affect the capacity of long-lived plants to compensate. Birches fully compensated for moderate browsing with an overall leaf biomass similar to unbrowsed birches but undercompensated under heavy browsing pressure. The main mechanism explaining compensation appears to be the conversion of short shoots into long shoots. The leaf area increased under heavy browsing pressure but only led to undercompensation. Fertilisation for two consecutive years did not influence the response of birch, thus we conclude that our results support the LRM hypothesis of equal tolerance under both high and low nitrogen availability. Our results highlight that the potential for compensatory growth in dwarf birch is surpassed under heavy browsing pressure independently of the fertilisation regime. In the context of the worldwide decline in caribou herds, the reduction in browsing pressure could act synergistically with global climate change to promote the current shrub expansion reported in subarctic regions.

## Introduction

The expansion of deciduous dwarf shrubs has been reported in many arctic and subarctic regions mainly in response to global climate change [1], [2]. Deciduous shrubs are a large component of caribou (*Rangifer tarandus*) diet in spring and early summer [3]–[5] when other resources are scarce and animals suffer from protein and mineral deficiency after the winter, gestation and parturition [6]. Some authors have demonstrated that consumption and trampling of dwarf shrubs can at least partially counteract the positive impact of increasing temperatures on Arctic shrubs [7], [8]. The arctic tundra is a low productivity environment compared to lower latitudes [9] where woody plants cannot escape large herbivores by outgrowing them.

In response to tissue removal by consumers, plants have evolved mechanisms to cope with herbivory such as avoidance and tolerance (sensu [10]). Tolerance is the capacity to maintain fitness following herbivory [11], [12] through reproduction or compensatory growth [13]. For example, *Betula pubescens* Ehrh, *B. pendula* Roth and *Pinus sylvestris* L. can compensate for lost tissues following browsing [14]. From the herbivore point of view, compensation could support the sustainability of resources within an animal home range. We are especially interested in how compensation is related to multiple browsing intensities, as the use of exclosure in many compensation studies only allows a dichotomic comparison [15] and could prevent the discovery of threshold in plant response [16].

**Figure 1:**
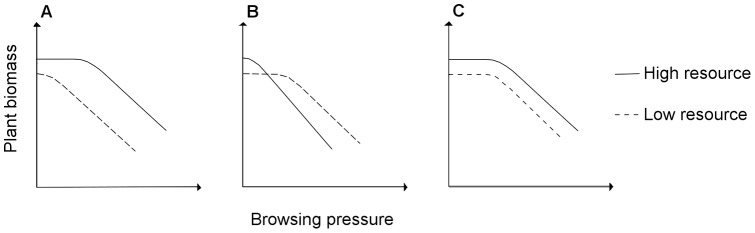
Conceptual representation of predictions from alternative hypotheses on compensation and resource availability integrating browsing pressure. The Compensatory Continuum Hypothesis (CCH, [17]) predicts higher compensation for tissues lost to herbivores in high resource environment up to a threshold browsing pressure and lower compensation in low ressource environment (A). The Growth-Rate Hypothesis (GRH, [18]) predicts higher compensation in low resource environment under low to moderate browsing pressure as plants have not yet attain their full growth potential (B). The prediction of equal tolerance between high and low resource environments of the Limiting Resource model (LRM, [23]) (C). In this situation, a resource is limiting plant growth while herbivory affects the use or acquisition of another non-limiting resource. As herbivory does not create a limitation in this alternate resource, compensation potential should be equal in high and low limiting resource environments. Intercepts are different between high and low resources, as we do not expect a similar plant biomass in those environments.

The compensatory response of plants may be influenced by the availability of resources such as water, nutrients, and light [11]. Two main hypotheses predict the degree of plant tolerance depending on resource availability such as water nutrient or light, with opposite predictions. The Compensatory Continuum Hypothesis (CCH, Fig. 1A) argues that the degree of compensatory growth is positively related to resource availability [17]. Persson, Bergström & Danell [14] found that a simulated heavy moose (*Alces alces*) browsing pressure stimulated *Betula pubescens* and *B. pendula* aboveground productivity in productive sites. At the opposite end of the gradient, the Growth Rate Hypothesis (GRH, Fig. 1B, [18], [19]) states that plants in low-resource environments have a superior ability for compensatory growth as they have not yet attain the full growth potential. For example, *Eucalyptus globulus* Labill. overcompensated under low water availability, while compensating to the biomass level of unbrowsed plant when water was supplied adequately [20]. The CCH, however, does not explicitly integrate a relationship between compensation potential and the amount of tissue removed while the GRH is not always tested with more than two browsing level as initially proposed by Hilbert et al [17]. Yet, increasing browsing has been shown both to decrease the level of compensation in *Salix viminalis* L. [21] or to increase compensation in *B. pubescens* and *B. pendula* (except under extreme browsing, [14]).

A meta-analysis by Hawkes and Sullivan [22] suggested that neither the CCH nor the GRH could explain the range of possible responses to herbivory. The Limiting Resource Model (LRM) was developed as a set of multiple hypotheses predicting tolerance depending on which resource is primarily affected by herbivory and which resource is limiting plant growth [23]. Predictions from the LRM are based on dichotomous keys [24] and can lead to predictions of the CCH and the GRH depending on the nature and availability of the limiting resources vs. the resource affected by browsing. For example, Baraza, Zamora & Hodar [25] found higher tolerance of three woody species under full light availability as predicted from the LRM when light was limiting tree growth and herbivory was damaging photosynthetic tissues.

Here, we investigated compensatory growth of the dwarf shrub *Betula glandulosa* Michx. in relation with browsing pressure and nitrogen availability; nitrogen usually being the most limiting nutrient in arctic tundra [9]. *B. glandulosa* is a dominant resource in the summer diet of caribou in eastern North America [3]–[5], especially for large caribou populations [3], [4] and recent studies report an increase in its abundance [26], [27]. We tested the predictions of the three alternative hypotheses presented before (CCH, GRH and LRM) and explicitly integrated the effect of browsing pressure in our predictions (Fig. 1) using a simulated browsing and nitrogen fertilisation experiment. According to the CCH, we predict higher tolerance (i.e. higher plant biomass) in high nutrient environments (Fig. 1A). Based on the GRH, we predict lower tolerance in low nutrient environments (Fig. 1B). The LRM predicts equal tolerance under both nutrient conditions (Fig. 1C) because damages from browsing mainly affect active meristems and carbon availability by removing carbon-fixing tissues, which are non-limiting resources [28]. Dwarf birch is a good candidate for compensation as it is heterophyllous; winter buds contain a definite number of early leaves, but late leaves are produced throughout the growing season [29]. They produce two types of shoots [30]. Long shoots are responsible for the current year growth and bear early and late leaves [29]. Short shoots bear only early leaves [29], but can convert to long shoots following herbivory [31]. Regardless of the hypothesis relating compensation to nutrient availability, we predict that compensation would mainly occur though the conversion of short shoots into long shoots following the response to the browsing treatment because of the high plasticity of birch in terms of shoot production [32].

## Materials and Methods

### Study area

Our study area is located near Deception bay (62.08′41′′N, 74.41′52′′O) Nunavik, Québec, Canada, within the summer range of the Rivière-aux-Feuilles caribou herd. The region is composed of intrusive tonalitic rocks [33]. According to the Circumpolar Arctic Vegetation Map [34], it is in the erect dwarf shrub tundra, on a mostly acidic soil. Species composition at study site is similar to the shrubby edges, thicket openings and open tundra described by Maycock and Matthews [35]. The entire territory is covered by continuous permafrost [36]. The first year of our study, July 2009, was warm and dry while 2010 was wetter and colder, resulting in a much smaller number of growing degree days (Table 1). It is possible that birches suffered a drought in 2009, as many ramets dried and died. Data from Katinniq (95 km south of Deception Bay) indicates 64.8 mm of rain for the months of June, July and August in 2009 compared to 164.6 mm in 2010 (V. Simoneau, pers. communication).

**Table 1:** Climatic conditions in July at Deception Bay, Nunavik, Canada.

Variables	2009 (95% CI)	2010 (95% CI)	2002–2007 (95% CI)
Temperature (°C)	13.9 (13.8; 14.0)	10.9 (10.8; 11.0)	9.3 (8.6; 10.0)*
Relative humidity (%)	56.5 (56.2; 56.7)	74.2 (74.0; 74.4)	-
Photosynthetically active radiations (μE)	1698 (1630; 1766)	1642 (1457; 1826)	-
Growing degree days over 5°C in July (days)	275	183	-
Precipitations June/July/August (mm)	64.8	164.6	184.7 (151.1; 218.2)*

* Temperatures in 2002–2007 are from Salluit (SILA network, CEN, Québec) and precipitations from Katinniq (V. Simoneau, pers. communication).

### Experimental design

**Figure 2:**
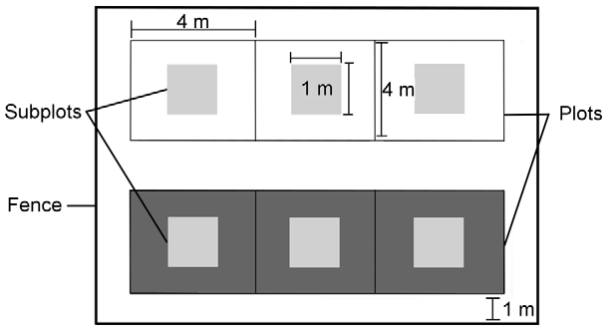
Representation of 1 block of an experiment testing the effects of fertilisation and browsing on compensation by *Betula glandulosa.* The experimental design was installed in 2009 at Deception Bay (Québec, Canada). Dark grey plots are fertilized with 10 g N/m^2^. The levels of the browsing treatment (0, 25 and 75% of available shoots >5 and ≤12 cm stripped of their leaves) were distributed randomly between subplots (pale grey). Numbers adjacent to dotted lines indicate their length. Each treatment was applied once a year.

We implemented a simulation experiment in 2009–10 with three levels of browsing pressure randomly allocated within two levels of fertilisation leading to a complete factorial split-block-split-block design with two split blocked factors, fertilisation and year [37]. Each combination of treatments was replicated in five blocks permanently enclosed by 12×26×1.5 m wire fences to exclude caribou (Fig. 2). All blocks were located on a south-west facing hill (slope between 1 and 11%) with their long axis parallel to the slope. Blocks were subdivided into 12 4×4 m plots, half of which received a temperature increment treatment included in a companion study that will not be discussed further here. The browsing treatment was applied to all ramets in a 1×1 m subplot within a 4×4 m secondary plot by manually stripping leaves [15] out of 0, 25% (i.e. every fourth shoot along a ramet) or 75% of available shoots. An available shoot was defined as a distal twig >5 cm and ≤12 cm) following Manseau [3]. As long shoots are not always >5 cm, the simulated browsing affected both long and short shoots. The simulated browsing treatment was applied from the moment we observed caribou stripping newly emerged birch leaves (1–5 July 2009, 23–28 June 2010). From hereafter, simulated browsing will be referred to as browsing. The six plots located downslope in each block were fertilized once a year by addition of 10 g nitrogen m^−2^ as granular urea [32] at the beginning of bud burst in early June (16-Jun-2009, 10-Jun-2010). Plots located upslope remained unfertilised. We cut trenches to permafrost level between plots to isolate birch clones. The slope affected the depth of snow (generalized linear model: F_10,23_ = 5.37, P = 0.0004); the highest accumulation of snow occurred at the top of the block and thus should not affect the humidity of fertilized vs. unfertilized plots as snow melt run down the slope.

We tested for nitrogen limitation using the N:P ratio in a composite sample of leaves from three unfertilised and unbrowsed plots [38]. N contents were estimated from dried and milled leaves, solubilised with the wet oxidation procedure of Parkinson & Allen [39] then processed with a flow injection analyzer (Quikchem 4000, Zellweger Analytics Inc., Lachat Instruments Division, Milwaykee, WI; [40]). P contents were prepared according to the same protocol and dosed with inductively coupled plasma optical emission spectrometry (Optima 4300DV, Perkin-Elmer Inc, Wellesley, MA). The mean N:P ratio was 11.1±4.1 (x^—^±95% CI), suggesting nitrogen limitation [38]. In average, leaves contained 2.80±0.06% of N and 0.24±0.01% of P.

We assessed the bioavailable nitrogen content of the soil with plant root simulators (PRS^TM^-probes, Western Ag Innovations Inc., Saskatoon, SK, Canada). The probes were inserted in the soil for two five-day burial periods beginning one week and one month after fertilisation in 2010. Fertilized plots had a bioavailable N rate higher than unfertilized plots (186±36 μg 10 cm^−2^ 5 days^−1^ vs 10±2 μg 10 cm^−2^ 5 days^−1^, mixed model, log-transformed: F_1,4_ = 174.8, P = 0.0002).

### Responses of dwarf birch

Compensatory growth was estimated from the production of aboveground non-reproductive tissues [22] i.e. total biomass [41], [42] and short and long shoot production [30], [43]. We estimated the biomass of birch leaves in subplots annually in early August using point intercept sampling with 25 systematic points within a 0.56 m^2^ frame [44], [45], leaving a small buffer on the sides of the subplots. For confirmation, we sampled 14 plots with 100 points. In the data of each of these plots, we resampled six replicates for each pin density between 10 to 90 points with a pace of 10, plus one sample at 100 points. This resulted in a total of 55 samples per plot. The relative number of hits per point was not related to the pin density (generalized linear model:, F_9,119_ = 0.5, P = 0.9).

We marked and followed three to seven ramets within subplots for two years to obtain a minimum of 50 shoots available for browsing. We followed ramets instead of an entire individual because of the clonal propagation of birches [46] and we isolated groups of browsed ramets by cutting trenches between plots. Each year, at the end of the growing season, we assessed the number of short and long shoots per ramet. We defined long and short shoots according to Ishihara and Kikuzawa [47]. In August 2010, we collected approximately 250 fresh leaves from available shoots per plot, pressed, dried and scanned them (HP Scanjet G4010, Mississauga). Images were transformed with the freeware Gimp v2.6 to maximum contrast and lower brightness for leaf differentiation. We used a modified blob detection program (Blobfinder.java, Greensted, 2009) to measure the number of pixels for each leaf and converted the area in pixels to an individual leaf area.

### Statistical analyses

We examined the effects of browsing and fertilisation on birch leaf biomass and structure (number of short and long shoots) using linear mixed models (MIXED procedure, [48]) with block and all interactions involving block entered as random factors and year as a repeated measure (Table 2). We visually assessed the normality of residuals and homogeneity of variance assumptions and applied transformations whenever required (square root and natural logarithm for long and short shoots, respectively). For leaf area in 2010, we used a generalized linear mixed model with a negative binomial distribution (GLIMMIX procedure, [49]). We conducted a posteriori mean comparisons with least square means (LSMEANS statement, [50]), by default this procedure analyses all pairwise differences. Data are presented as untransformed x^—^±95% CI. All statistical analyses were performed with SAS 9.2 [51] with α = 0.05.

**Table 2:** Productivity and structural responses of *Betula glandulosa* in response to simulated browsing (0, 25% and 75% of shoots ≥5 cm stripped of their leaves) and fertilisation (natural level and addition of 10 g N m^−2^) during two years (2009–2010).

Sources of variation	*Df (num, den)*	Leaf biomass*		Leaf area		Number of short shoots		Number of long shoots	
		F	P	F	P	F	P	F	P
Fertilisation	1,4	0.1	0.8	0.8	0.4	0.2	0.7	0.5	0.5
Browsing	2,16	**5.7**	**0.01**	**3.7**	**0.05**	**6.9**	**0.007**	**9.5**	**0.002**
Fertilisation × Browsing	2,16	1.3	0.3	0.5	0.6	0.1	0.9	0.08	0.9
Year	1,24	0.03	0.9	-	-	0.3	0.6	**52.4**	**<0.0001**
Fertilisation × Year	1,24	1.0	0.3	-	-	4.2	0.051	0.5	0.5
Browsing × Year	2,24	1.0	0.4	-	-	**6.4**	**0.006**	3.0	0.07
Fertilisation × Browsing × Year	2,24	1.1	0.4	-	-	1.4	0.3	0.6	0.6

ANOVA results are based on linear mixed models with block (n = 5) and all interactions involving block as random factors and year as a repeated measure, except for leaf area, for which the analysis was only performed in 2010. Numbers in bold are statistically significant (α = 0.05).

* Leaf biomass is estimated from point intercept on leaves.

## Results

### Biomass and leaf area

**Figure 3:**
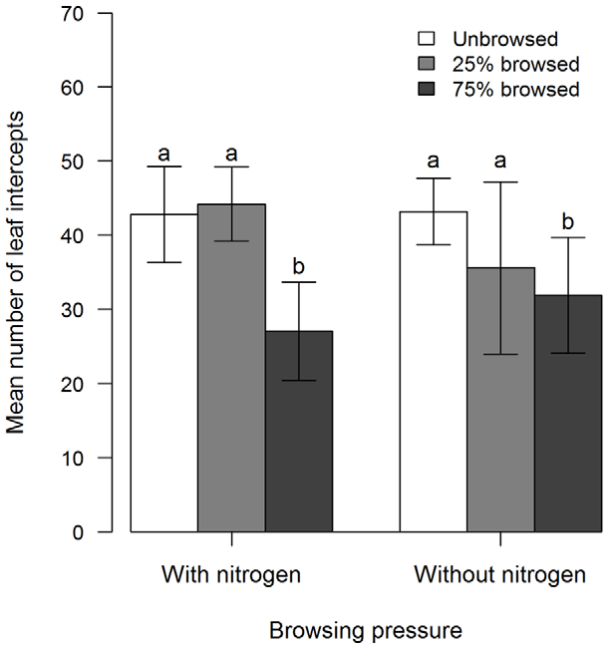
Compensation responses of *Betula glandulosa* Michx. under experimental variation in resource availability and browsing pressure. The similar number of leaf intercepts (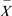
±95% CI), a proxy for leaf biomass, in unbrowsed and moderately browsed plots indicates exact compensation while no compensation or undercompensation occurred in the heavily browsed one independently of resource availability for the plants as predicted by the Limiting Resource Model. Leaf point interception was performed based on 25 points in a 0.56 m^2^ frame in August after two years of simulated caribou browsing and fertilisation near Deception Bay (Nunavik, Québec, Canada). The means shown are for 2009 and 2010 pooled because year did not influence the response to browsing. The analysis was performed using a linear mixed model with block (n = 5) and all interactions involving block as random factors and year as a repeated measure. Different letters over bars indicate a posteriori least square mean differences (α = 0.05).

Browsing was the only experimental treatment influencing birch leaf biomass and leaf area (Table 2). For both years and fertilisation levels, the leaf biomass of *B. glandulosa* in moderately browsed plots recovered to the level estimated in unbrowsed plots (t_16_ = 0.7, P = 0.5) while declining by about 30% in plots under heavy browsing pressure (unbrowsed vs heavily browsed: t_16_ = 3.2, P = 0.005; moderately vs heavily browsed t_16_ = 2.5, P = 0.02; Table 2 and Fig. 3). Individual leaf area was higher for heavily browsed than unbrowsed *B. glandulosa* (134±2 mm^2^ and 115±2 mm^2^ respectively, t_24_ = −2.69, P = 0.02), but comparable to moderately browsed ones (heavy vs moderate: 123±2 mm^2^, t_24_ = −1.7, P = 0.1; Table 2).

### Structure: short and long shoots

**Figure 4:**
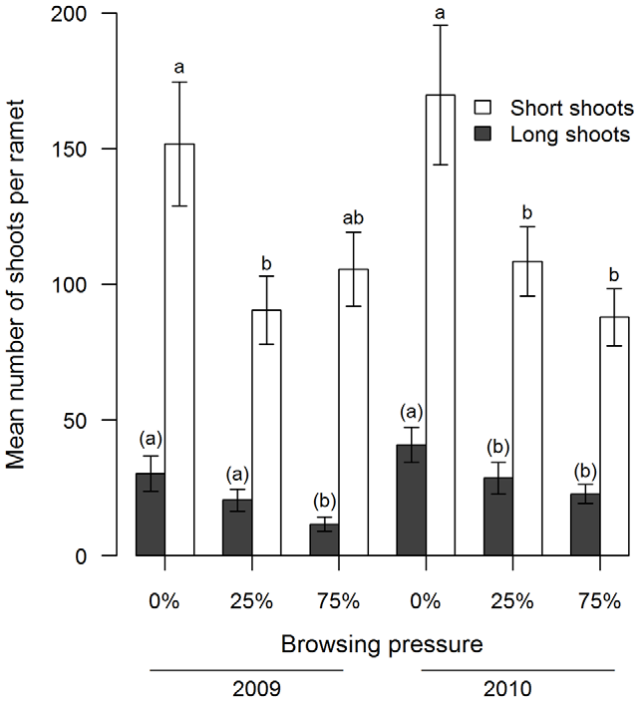
Growth allocation in short and long shoots in *Betula glandulosa* Michx. The similar ratio of *Betula glandulosa* Michx. short to long shoots (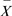
±95% CI) in unbrowsed and moderately browsed plots in 2009 and under all browsing pressure in 2010 suggests conversion of short shoots into long shoots. The number of short shoots and long shoots was estimated on marked ramets in August after two years of simulated caribou browsing and fertilisation near Deception Bay (Nunavik, Québec, Canada). The analysis was performed using a linear mixed model with block (n = 5) and all interactions involving block as random factors and year as a repeated measure. Transformations were applied, square root and natural logarithm for long and short shoots, respectively. Means and CI presented are untransformed. Different letters over bars indicate a posteriori least square mean differences in simulated browsing level for each year on transformed data; (α = 0.05). For long shoots, the a posteriori test was performed on transformed data, on a nearly significant interaction, (α = 0.05).

In 2009, moderately browsed ramets had 40% fewer short shoots than unbrowsed ones (t_24_ = 2.7, P = 0.01; Table 2, Fig. 4), while heavily browsed ramets could not be discriminated from unbrowsed ramets in terms of short shoot numbers (t_24_ = 1.8, P = 0.09). The number of short shoots in heavily browsed ramets declined by 48% compared to unbrowsed ramets in 2010 (t_24_ = 4.5, P = 0.0002) and moderately browsed ramets had 36% fewer short shoots than unbrowsed ones (t_24_ = 2.8, P = 0.01). In both years, moderately browsed and heavily browsed ramets presented a similar number of short shoots (2009: t_24_ = −0.9, P = 0.4; 2010: t_24_ = 1.7, P = 0.1; Table 2, Fig. 4).

The abundance of long shoots decreased by 51% under heavy browsing pressure compared to unbrowsed ramets (t_24_ = 4.4, P = 0.0005), but increased from 21±3 shoots in 2009 to 31±3 in 2010 independently of treatment (Table 2). There was also a statistical trend for an interaction between browsing and year (Fig. 4). In 2009, moderately browsed and unbrowsed ramets had a similar number of long shoots (t_24_ = 1.5, P = 0.1), while it was statistically different in 2010 (t_24_ = 2.5, P = 0.02). Moderately and heavily browsed ramets, that had a different number of long shoots in 2009 (t_24_ = 3.0, P = 0.006), were similar in 2010 (t_24_ = 1.0, P = 0.4).

The abundance of short shoots tended to increase in unfertilised plots from 110±14 in 2009 to 126±14 in 2010 (t_24_ = −1.86, P = 0.08) while remaining stable in fertilised plots (122±17 to 118±19; t_24_ = 1.05, P = 0.3; Table 2). Long shoot abundance was unrelated to fertilisation (Table 2).

## Discussion

Our simulation experiment combining a gradient of browsing pressure and two fertilisation levels revealed compensatory growth of *B. glandulosa* under moderate browsing pressure independently of nitrogen availability. This is in accordance with the predictions of the limiting resource model when herbivores affect a non-limiting alternate resource (Fig. 1C). Even if apical meristems and carbon are not limiting compared to nutrient availability, removal of 75% of the shoots available to browsing led to under-compensation.

Short shoots were as abundant in heavily browsed as in moderately browsed birches, at least in one year, despite the much higher removal of short shoots in the former. Similarly, long shoots were as abundant in unbrowsed birches as in moderately browsed birches in 2009. We did not measure individual shoot conversion but these results suggest the conversion of short shoots into long shoots at moderate browsing pressure. Lehtilä et al. [52] demonstrated that a small percentage of short shoots can convert into long shoots and a subsequent experiment demonstrated that this conversion can be triggered by the removal of apical buds [31]. In 2010, moderately browsed and heavily browsed birches presented the same number of short and long shoots and thus, we cannot tell if conversion occurred. Dormant meristems could have convert into long shoot. However, we did not observe many dormant meristems and a bud demography study on a closely related birch species [52] suggests that dormant buds mostly convert to short shoots.

Plasticity in shoot production could provide a mechanistic explanation for the full compensation of leaf biomass at moderate browsing pressure. The conversion of short shoots into long shoots allowed the leaf biomass to remain similar at unbrowsed and intermediate browsing pressure. Long shoots produce both early and late leaves [29], allowing an indefinite production of leaves throughout the growing season and thus, an increase in leaf biomass. The conversion of short shoots into long shoots is probably triggered by the release of apical dominance [32], because caribou browsing usually damages terminal buds [3]. This mechanism has been documented in some birch species [29], [52], in response to browsing [31] and to fertilisation and warming in *B. nana* L. [32]. Alternatively, compensation could result from the increased of individual leaf area. Under heavy browsing pressure, birches apparently allocated their residual resources to leaf expansion rather than the production of new leaves even though this could not fully compensate for lost tissues. Still, it demonstrated the plasticity of dwarf birch in response to browsing. Increases in leaf area following summer herbivory have also been recorded in *B. pendula* in a 1 year simulated browsing experiment [53] and following bud removal in *Betula pubescens* ssp. *tortuosa* (Ledeb.) Nyman [54]. Compensation could also occur from sprouting of new ramets, but our data do not allow us to test this hypothesis.

We found an effect of year for short and long shoot numbers, suggesting a change in birch growth between a dry (2009) and average (2010) summer (Table 2). Because of the low precipitation at our study site, water availability may be a factor limiting birch growth. In 2010, unbrowsed birches had a high production of long shoots that could not be matched at moderate browsing pressure. The similar proportion of short shoots at all browsing intensities during this productive year suggests that conversion to long shoots could have occurred in highly browsed shrubs. This conversion did not result in leaf biomass compensation, probably because of a lower number of leaves on the long shoots. Kaitaniemi, Neuvonen & Nyyssonen [55] demonstrated that heavy defoliation can reduce long shoots length in *Betula pubescens*.

Opposite to the predictions of the CCH and GRH hypotheses but according to the LRM, we could not detect an effect of fertilisation on birch compensation. Similarly, Melnychuk and Krebs [56] found that snowshoe hare (*Lepus americanus*) abundance affected *B. glandulosa* growth more than fertilisation. However, Gough, Ramsey & Johnson [57] found fertilisation effects on *B. nana* growth in a 9-year experiment and Bret-Harte et al. [32] found increases in biomass of *B. nana* after 7 years of fertilisation. The level of N added to fertilised plots did increase the availability of N for plants. Using the same quantity of nitrogen added, Hobbie, Gough & Shaver [58] observed increased cover of *B. nana* 2 years following fertilisation. *B. nana* is a closely related species, hard to distinguish from *B. glandulosa* [59] and no study to our knowledge compared their response to fertilisation or browsing. Nevertheless, there could be a time lag in the response to fertilisation. The high variance in leaf biomass responses under moderately browsed and unfertilised plots led us to conclude that there was no effect of fertilisation on the growth of birch but higher statistical power could have change our conclusion in favor of the CCH. Size and age of the plants may introduce variability in the compensatory response reducing our capacity to detect an effect of fertilisation but these dependencies are poorly known [60].

Viewing LRM as a set of hypotheses as opposed to one model implies testing distinct hypotheses depending on prior assumptions [61]. For instance, equal tolerance in both high and low resource environments as we observed is predicted if the three following assumptions are met. First, a focal resource needs to be limiting. This was confirmed by the low N:P ratio at our study site. Second, we assumed that browsing primarily affected use/acquisition of carbon or apical meristems, and not nitrogen, based on results of Palacio et al. [62]. Those results showed that leaf nitrogen concentration was unaltered by repeated summer browsing in *B. pubescens* while leaf carbon concentration decreased following short-term browsing. They also observed a decrease in C in leaves of *B. pubescens* following short-term browsing, confirming that browsing affects C dynamics. Damages to apical meristems by leaf stripping were also confirmed by observation of caribou browsing [3]. If N was the limiting resource in our system, we would have change the prediction to higher compensation in high resource environment but our results do not fit with this prediction. Third, carbon availability or the number of apical meristems should not limit plant fitness in fertilised birches. In arctic ecosystems, plants are usually rich in carbohydrates [63]. Birches also presented many short shoot meristems that could convert into long shoots, and thus cannot be considered limiting [31], [32].

Wise and Abrahamson [23] stressed the importance of using factorial experiments with at least two levels of browsing when testing the LRM. Yet, using only two browsing level (high and none), we would have miss the compensation at moderate browsing intensity. We recommend using multiple levels of herbivory when studying tolerance in woody plants. Because of their modular structure, woody plants may have more diverse responses than monocotyledons that are either browse of not. Woody plants modules can be relatively independent and there is a difference in effect size between a browsing event affecting one module and another affecting all modules of a woody plant [60]. By integrating varying browsing pressure into the LRM, we would improve its predictive abilities for woody species.

## Conclusions

We demonstrated a compensatory response of *B. glandulosa* under moderate browsing pressure mainly through adjustments in the production of short vs. long shoots. Tolerance was unaffected by the soil nitrogen status after two years of simulation. Because birch leaf production is highly plastic and exhibit compensation at moderate browsing pressure, it could provide a reliable forage resource for caribou. We still have to demonstrate whether higher leaf biomass translates into higher forage availability and what effect browsing has on forage nutritive quality. Lower forage availability could occur even under full compensation if the structure of the shrubs is modified. We also demonstrated the usefulness of the LRM, when assumptions are met, and we integrated the effect of browsing pressure in our predictions. On the other hand, we tested only one focal resource but birches could also be limited by water availability. Although *B. glandulosa* seems tolerant to periodic droughts, xeric drainage can be detrimental to the species [59]. Our results showed that caribou can decrease birch leaf biomass, thus with recent declines in caribou populations worldwide [64] the subsequent decline in browsing pressure could benefit the current shrub expansion [1].
